# HEARING LOSS AS A MODIFIABLE RISK FACTOR FOR DEMENTIA IN UK BIOBANK

**DOI:** 10.1093/geroni/igad104.0229

**Published:** 2023-12-21

**Authors:** Jure Mur, Anja Leist

**Affiliations:** The University of Edinburgh, Edinburgh, Scotland, United Kingdom; University of Luxembourg, Esch-sur-Alzette, Luxembourg

## Abstract

Age-related hearing loss is a predictor of cognitive impairment and dementia. The mechanisms of the observed associations are unclear but could involve depletion of cognitive reserve, the occupation of cognitive resources, and lack of sensory input to the brain. Hearing loss may therefore represent a preventable or treatable causative risk factor for dementia. Indeed, many studies report on lower rates of cognitive decline among hearing aid users when compared to non-users. However, most investigations to date have been observational or with relatively short follow-ups, thus precluding clear interpretations of the underlying mechanism. In our study using UK Biobank data with n=502,386 participants followed up from ~2008 to 2021, we emulate a randomised clinical trial to probe the role of initiating the use of hearing aids in people with conductive hearing loss or with self-reported hearing difficulties in the diagnosis dementia. Diagnosis of dementia was ascertained through hospital and death records, and self-reports in such a way as to increase the positive predictive value. Among participants with hearing loss or hearing difficulties, 2,914 (2.1%) were diagnosed with dementia, compared to 4,982 (1.4%) in those without hearing loss or hearing difficulties. Additional results on the relationship between hearing aid use and dementia, and stratified analyses by sex/gender and ethnicity will be presented. We discuss the modifiability of hearing loss and recommend studies with longer follow-ups to determine the efficacy of hearing loss interventions in reducing the risk of cognitive decline and dementia.

